# Supplementary material for: Comparative genomics provides new insights into the diversity, physiology, and sexuality of the only industrially exploited tremellomycete: Phaffia rhodozyma
Source: BMC Genomics. 2016 Nov 9;17:901. doi: 10.1186/s12864-016-3244-7 (PMC5103461; doi:10.1186/s12864-016-3244-7)
Supplement: Additional file 6: — List of orphan genes with links to PFAM (related to Additional file 1: Table S1). (ZIP 1428 kb) [file 12864_2016_3244_MOESM6_ESM.zip › PFAM_UNIQUE/G03875_P.html]

#                                                                            --- full sequence --- -------------- this domain -------------   hmm coord   ali coord   env coord
  
# target name        accession   tlen query name           accession   qlen   E-value  score  bias   #  of  c-Evalue  i-Evalue  score  bias  from    to  from    to  from    to  acc description of target
  
#------------------- ---------- ----- -------------------- ---------- ----- --------- ------ ----- --- --- --------- --------- ------ ----- ----- ----- ----- ----- ----- ----- ---- ---------------------
  
G03875\_P             -             76 DUF4231              PF14015.1    112     0.015   14.0   0.7   1   1   1.5e-05     0.018   13.7   0.7    21    75     9    74     3    75 0.63 - Protein of unknown function (DUF4231)  
G03875\_P             -             76 DUF4244              PF14029.1     57     0.085   10.7   2.1   1   1   2.6e-05      0.16    9.8   2.1    25    38    57    70    54    73 0.89 - Protein of unknown function (DUF4244)
